# (*S*)-6-{[(*S*)-2,2-Dimethyl-1,3-dioxolan-4-yl]meth­yl}-5,5-difluoro-5,6-dihydro-2*H*-pyran-2-one

**DOI:** 10.1107/S1600536809028256

**Published:** 2009-07-25

**Authors:** Zengsheng Yin, Xiangjun Deng, Rongxing Yao, Hongqi Li, Pinqiao Zhao

**Affiliations:** a Key Laboratory of Science & Technology of Eco-Textiles, Ministry of Education, College of Chemistry, Chemical Engineering and Biotechnology, Donghua University, Shanghai 201620, People’s Republic of China; b Key Laboratory of Organofluorine Chemistry, Shanghai Institute of Organic Chemistry, Chinese Academy of Science, Shanghai 200032, People’s Republic of China

## Abstract

The title compound, C_11_H_14_F_2_O_4_, is a γ,γ-gem-difluorinated α,β-unsaturated δ-lactone. The dioxolane five-membered ring and the lactone ring adopt half-chair conformations. There are two inter­molecular C—H⋯O inter­actions involving the carbonyl group as an acceptor which stabilize the crystal structure.

## Related literature

For related synthetic procedures, see: Borjesson & Welch (1992 ▶); Dardonville & Gilbert (2003 ▶); Gaunt *et al.* (2003 ▶); Saito *et al.* (1992 ▶); You *et al.* (2006 ▶).
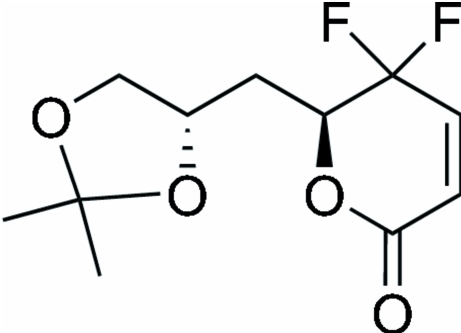


## Experimental

### 

#### Crystal data

C_11_H_14_F_2_O_4_
*M* *_r_* = 248.22
Orthorhombic, 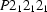

*a* = 5.8003 (8) Å
*b* = 7.8135 (11) Å
*c* = 25.977 (4) Å
*V* = 1177.3 (3) Å^3^
*Z* = 4
Mo *K*α radiation
μ = 0.13 mm^−1^
*T* = 293 K
0.51 × 0.48 × 0.26 mm

#### Data collection

Bruker SMART APEX CCD area-detector diffractometer
Absorption correction: multi-scan (*SADABS*; Bruker, 2001 ▶) *T*_min_ = 0.761, *T*_max_ = 1.000 (expected range = 0.736–0.968)
6682 measured reflections
1455 independent reflections
1261 reflections with *I* > 2σ(*I*)
*R*_int_ = 0.131

#### Refinement

*R*[*F* ^2^ > 2σ(*F* ^2^)] = 0.054
*wR*(*F* ^2^) = 0.134
*S* = 1.00
1455 reflections
173 parameters
H atoms treated by a mixture of independent and constrained refinement
Δρ_max_ = 0.25 e Å^−3^
Δρ_min_ = −0.32 e Å^−3^

### 

Data collection: *SMART* (Bruker, 2001 ▶); cell refinement: *SAINT* (Bruker, 2001 ▶); data reduction: *SAINT*; program(s) used to solve structure: *SHELXS97* (Sheldrick, 2008 ▶); program(s) used to refine structure: *SHELXL97* (Sheldrick, 2008 ▶); molecular graphics: *SHELXTL* (Sheldrick, 2008 ▶); software used to prepare material for publication: *SHELXTL*.

## Supplementary Material

Crystal structure: contains datablocks global, I. DOI: 10.1107/S1600536809028256/gk2220sup1.cif

Structure factors: contains datablocks I. DOI: 10.1107/S1600536809028256/gk2220Isup2.hkl

Additional supplementary materials:  crystallographic information; 3D view; checkCIF report

## Figures and Tables

**Table 1:** Hydrogen-bond geometry (Å, °)

*D*—H⋯*A*	*D*—H	H⋯*A*	*D*⋯*A*	*D*—H⋯*A*
C8—H8⋯O1^i^	0.95 (3)	2.66 (3)	3.578 (4)	161 (2)
C6—H6⋯O1^ii^	0.96 (3)	2.44 (3)	3.318 (4)	151 (2)

Symmetry codes: (i) 
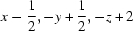
; (ii) 
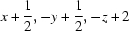
.

**Fig. 1.**
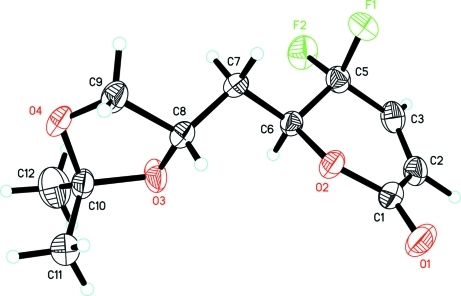
A view of the molecule of the title compound. Displacement ellipsoids are drawn at the 30% probability level and H atoms are shown as small spheres of arbitrary radii.

**Fig. 2.**
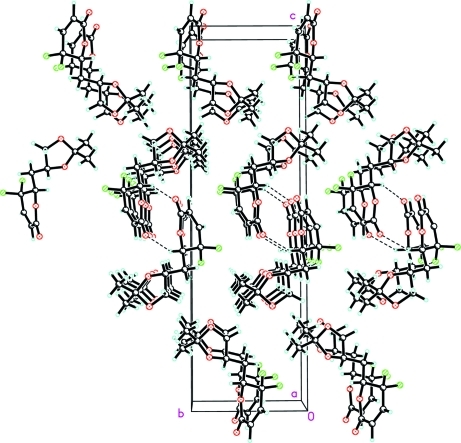
The crystal structure of the title compound, viewed along a axis. Dashed lines indicate the hydrogen bond interactions.

**Fig. 3.**
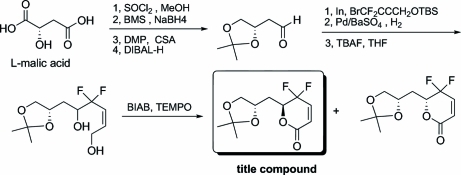
The scheme of synthesis of the title compound.

## supplementary crystallographic information

### Comment

α,β-Unsaturated δ-lactone is a common structural unit of natural products with bioactivity. The structure-activity relationship (SAR) reveals that the unsaturated lactone often plays a key role in the bioactivity. The reason may be that the unsaturated lactone is an excellent potential Michael acceptor for natural nucleophiles such as the amino-acid residues. The title compound is an γ,γ-gem-difluorinated α,β-unsaturated δ-lactone, a better Michael acceptor for the electron-withdrawing of the difluoromethylene group. So it is a useful intermediate for synthesis of the fluorine-containing analogues of natural product with potential bioactivity. The title compound was prepared from *L*-malic acid according to the method developed by our group (You *et al.*, 2006) and other groups (Borjesson *et al.*, 1992; Dardonville *et al.*, 2003; Gaunt *et al.*, 2003; Saito *et al.*, 1992). Our interest is focused on the changes caused by introducing difluoromethylene group into the lactone ring. Here we report the crystal structure of the title compound.

The absolute configuration of the title compound was determined by the known chirality of the C8 derived from the starting material, *L*-malic acid. All bond lengths and angles in the lactone ring are within normal ranges. The dioxolane five-membered ring and the lactone ring both adopt a half-chair conformation. Intermolecular interactions C6—H6···O1 and C8—H6···O1 arrange the moleculesd in a head-to-head fashion (see Fig. 2).

### Experimental

For the reaction scheme see Figure 3. To a solution of (*Z*)-6-((*S*)-2,2-dimethyl-1,3-dioxolan-4-yl)-4,4- difluorohex-2-ene-1,5-diol in CH_2_Cl_2_ was added bis-acetoxyiodobenzene (3 eq) and 2,2,6,6-tetramethyl piperidinooxy (0.1 eq) at room temperature. After stirring for 3 h, the reaction was quenched with saturated solution of Na_2_S_2_O_3_ and extracted with CH_2_Cl_2_. The combined organic extracts were washed with saturated solution of NaHCO_3_, NH_4_Cl, brine, dried over anhydrous MgSO_4_, filtered and concentrated. The residue was purified by flash chromatography on silica gel (petroleum ether / ethyl acetate = 10:1) to afford the title compound. Crystals suitable for X-ray structural analysis were obtained by slow evaporation of a solution in actone and petroleum ether (1:1, *v*/*v*).

### Refinement

All H atoms could be located in a difference Fourier map. The H atoms from the piran-2-one fragment and the methine H8 atom were fully refined. The remaining H atoms were placed in calculated positions (C-H = 0.97-0.98 Å) and refined using a riding model approximation with *U*_iso_(H) = 1.2*U*_eq_(C) or *U*_iso_(H) = 1.5*U*_eq_(methyl C). Friedel pairs were merged as no significant anomalous scattering effects were observed. The absolute configuration was related to a known chirality (S) of the dioxolane C8 atom. The high R_int_ value results from a poor quality of the measured crystal.

### Crystal data

**Table:**

C_11_H_14_F_2_O_4_	*D*_x_ = 1.400 Mg m^−^^3^
*M**_r_* = 248.22	Melting point: 351 K
Orthorhombic, *P*2_1_2_1_2_1_	Mo *K*α radiation, λ = 0.71073 Å
Hall symbol: P 2ac 2ab	Cell parameters from 2723 reflections
*a* = 5.8003 (8) Å	θ = 2.7–27.7°
*b* = 7.8135 (11) Å	µ = 0.13 mm^−^^1^
*c* = 25.977 (4) Å	*T* = 293 K
*V* = 1177.3 (3) Å^3^	Prismatic, colorless
*Z* = 4	0.51 × 0.48 × 0.26 mm
*F*(000) = 520	

### Data collection

**Table:**

Bruker SMART APEX CCD area-detector diffractometer	1455 independent reflections
Radiation source: fine-focus sealed tube	1261 reflections with *I* > 2σ(*I*)
graphite	*R*_int_ = 0.131
Detector resolution: 0 pixels mm^-1^	θ_max_ = 26.5°, θ_min_ = 1.6°
φ and ω scans	*h* = −7→7
Absorption correction: multi-scan (*SADABS*; Bruker, 2001)	*k* = −9→9
*T*_min_ = 0.761, *T*_max_ = 1.000	*l* = −22→32
6682 measured reflections	

### Refinement

**Table:**

Refinement on *F*^2^	Secondary atom site location: difference Fourier map
Least-squares matrix: full	Hydrogen site location: inferred from neighbouring sites
*R*[*F*^2^ > 2σ(*F*^2^)] = 0.054	H atoms treated by a mixture of independent and constrained refinement
*wR*(*F*^2^) = 0.134	*w* = 1/[σ^2^(*F*_o_^2^) + (0.0757*P*)^2^ + 0.0067*P*] where *P* = (*F*_o_^2^ + 2*F*_c_^2^)/3
*S* = 1.00	(Δ/σ)_max_ = 0.001
1455 reflections	Δρ_max_ = 0.25 e Å^−^^3^
173 parameters	Δρ_min_ = −0.32 e Å^−^^3^
0 restraints	Extinction correction: *SHELXL97* (Sheldrick, 2008), Fc^*^=kFc[1+0.001xFc^2^λ^3^/sin(2θ)]^-1/4^
Primary atom site location: structure-invariant direct methods	Extinction coefficient: 0.024 (5)

### Special details

**Table:**

Geometry. All e.s.d.'s (except the e.s.d. in the dihedral angle between two l.s. planes) are estimated using the full covariance matrix. The cell e.s.d.'s are taken into account individually in the estimation of e.s.d.'s in distances, angles and torsion angles; correlations between e.s.d.'s in cell parameters are only used when they are defined by crystal symmetry. An approximate (isotropic) treatment of cell e.s.d.'s is used for estimating e.s.d.'s involving l.s. planes.
Refinement. Refinement of *F*^2^ against ALL reflections. The weighted *R*-factor *wR* and goodness of fit *S* are based on *F*^2^, conventional *R*-factors *R* are based on *F*, with *F* set to zero for negative *F*^2^. The threshold expression of *F*^2^ > σ(*F*^2^) is used only for calculating *R*-factors(gt) *etc*. and is not relevant to the choice of reflections for refinement. *R*-factors based on *F*^2^ are statistically about twice as large as those based on *F*, and *R*- factors based on ALL data will be even larger.

### Fractional atomic coordinates and isotropic or equivalent isotropic displacement parameters (Å^2^)

**Table:**

	*x*	*y*	*z*	*U*_iso_*/*U*_eq_	
O1	0.3246 (5)	0.3829 (4)	1.04388 (8)	0.0741 (7)	
O2	0.3476 (3)	0.4190 (2)	0.96034 (7)	0.0476 (5)	
O3	0.3431 (4)	0.1554 (3)	0.85332 (9)	0.0639 (7)	
O4	0.1373 (4)	0.1699 (3)	0.78051 (8)	0.0644 (6)	
F1	0.5404 (4)	0.7375 (2)	0.92182 (8)	0.0666 (6)	
F2	0.8095 (3)	0.5889 (3)	0.88536 (7)	0.0720 (6)	
C1	0.4418 (5)	0.4245 (4)	1.00830 (11)	0.0517 (7)	
C2	0.6747 (6)	0.4915 (4)	1.01335 (13)	0.0570 (8)	
C3	0.7764 (5)	0.5731 (4)	0.97527 (13)	0.0559 (8)	
C5	0.6553 (4)	0.5857 (4)	0.92469 (11)	0.0473 (6)	
C6	0.4949 (4)	0.4373 (3)	0.91582 (10)	0.0393 (6)	
C7	0.3425 (5)	0.4559 (3)	0.86946 (11)	0.0436 (6)	
H7A	0.4376	0.4761	0.8394	0.052*	
H7B	0.2433	0.5546	0.8741	0.052*	
C8	0.1945 (5)	0.2985 (4)	0.86032 (11)	0.0468 (6)	
C9	0.0516 (6)	0.3041 (4)	0.81085 (13)	0.0612 (8)	
H9A	−0.1107	0.2872	0.8183	0.073*	
H9B	0.0706	0.4131	0.7935	0.073*	
C10	0.2494 (5)	0.0526 (4)	0.81349 (11)	0.0529 (7)	
C11	0.0790 (8)	−0.0735 (4)	0.83574 (15)	0.0712 (9)	
H11A	0.0221	−0.1464	0.8088	0.107*	
H11B	−0.0473	−0.0124	0.8510	0.107*	
H11C	0.1539	−0.1418	0.8615	0.107*	
C12	0.4412 (7)	−0.0334 (6)	0.7860 (2)	0.0917 (14)	
H12A	0.5456	0.0512	0.7728	0.137*	
H12B	0.3803	−0.0998	0.7581	0.137*	
H12C	0.5218	−0.1073	0.8094	0.137*	
H2	0.758 (6)	0.483 (5)	1.0503 (15)	0.070 (10)*	
H3	0.936 (7)	0.614 (5)	0.9775 (14)	0.070 (10)*	
H6	0.588 (4)	0.335 (3)	0.9143 (11)	0.038 (7)*	
H8	0.084 (5)	0.278 (3)	0.8868 (12)	0.039 (7)*	

### Atomic displacement parameters (Å^2^)

**Table:**

	*U*^11^	*U*^22^	*U*^33^	*U*^12^	*U*^13^	*U*^23^
O1	0.0934 (15)	0.0921 (17)	0.0369 (11)	−0.0240 (15)	0.0040 (11)	0.0066 (13)
O2	0.0503 (9)	0.0605 (11)	0.0319 (9)	−0.0088 (9)	−0.0024 (7)	0.0022 (9)
O3	0.0785 (13)	0.0511 (11)	0.0622 (14)	0.0125 (11)	−0.0381 (12)	−0.0168 (12)
O4	0.0925 (15)	0.0675 (13)	0.0331 (10)	0.0026 (13)	−0.0193 (11)	0.0025 (11)
F1	0.0884 (12)	0.0420 (8)	0.0694 (13)	0.0005 (8)	−0.0035 (10)	−0.0024 (10)
F2	0.0613 (10)	0.0915 (13)	0.0633 (12)	−0.0178 (10)	0.0215 (9)	−0.0051 (11)
C1	0.0662 (15)	0.0513 (15)	0.0377 (14)	−0.0059 (14)	−0.0075 (13)	0.0004 (14)
C2	0.0676 (17)	0.0553 (15)	0.0481 (16)	−0.0037 (15)	−0.0169 (15)	−0.0088 (15)
C3	0.0468 (14)	0.0617 (17)	0.0591 (18)	−0.0077 (13)	−0.0064 (13)	−0.0158 (16)
C5	0.0487 (12)	0.0468 (14)	0.0465 (15)	−0.0032 (12)	0.0073 (12)	−0.0055 (13)
C6	0.0424 (12)	0.0418 (13)	0.0336 (13)	0.0014 (10)	0.0024 (10)	−0.0028 (12)
C7	0.0532 (13)	0.0448 (13)	0.0328 (12)	0.0013 (11)	−0.0011 (11)	0.0044 (11)
C8	0.0569 (14)	0.0484 (14)	0.0352 (13)	0.0006 (12)	−0.0093 (13)	0.0018 (13)
C9	0.0747 (17)	0.0619 (18)	0.0470 (17)	0.0064 (15)	−0.0250 (16)	−0.0014 (16)
C10	0.0641 (15)	0.0534 (16)	0.0414 (15)	0.0008 (13)	−0.0142 (13)	−0.0082 (14)
C11	0.096 (2)	0.0593 (19)	0.058 (2)	−0.0092 (18)	−0.0136 (18)	−0.0016 (18)
C12	0.084 (2)	0.094 (3)	0.098 (3)	0.008 (2)	0.006 (2)	−0.031 (3)

### Geometric parameters (Å, °)

**Table:**

O1—C1	1.193 (4)	C6—H6	0.96 (3)
O2—C1	1.361 (3)	C7—C8	1.518 (4)
O2—C6	1.445 (3)	C7—H7A	0.9700
O3—C10	1.418 (3)	C7—H7B	0.9700
O3—C8	1.424 (4)	C8—C9	1.530 (4)
O4—C9	1.402 (4)	C8—H8	0.95 (3)
O4—C10	1.414 (4)	C9—H9A	0.9700
F1—C5	1.362 (3)	C9—H9B	0.9700
F2—C5	1.358 (3)	C10—C12	1.482 (5)
C1—C2	1.455 (5)	C10—C11	1.511 (5)
C2—C3	1.316 (5)	C11—H11A	0.9600
C2—H2	1.08 (4)	C11—H11B	0.9600
C3—C5	1.493 (4)	C11—H11C	0.9600
C3—H3	0.98 (4)	C12—H12A	0.9600
C5—C6	1.505 (3)	C12—H12B	0.9600
C6—C7	1.501 (4)	C12—H12C	0.9600
			
C1—O2—C6	119.5 (2)	O3—C8—C7	108.3 (2)
C10—O3—C8	107.82 (19)	O3—C8—C9	104.1 (2)
C9—O4—C10	107.9 (2)	C7—C8—C9	114.5 (2)
O1—C1—O2	118.2 (3)	O3—C8—H8	111.6 (17)
O1—C1—C2	123.9 (3)	C7—C8—H8	113.8 (17)
O2—C1—C2	117.8 (3)	C9—C8—H8	104.2 (17)
C3—C2—C1	121.5 (3)	O4—C9—C8	105.0 (2)
C3—C2—H2	120 (2)	O4—C9—H9A	110.7
C1—C2—H2	118 (2)	C8—C9—H9A	110.7
C2—C3—C5	118.9 (3)	O4—C9—H9B	110.7
C2—C3—H3	122 (2)	C8—C9—H9B	110.7
C5—C3—H3	118 (2)	H9A—C9—H9B	108.8
F2—C5—F1	105.4 (2)	O4—C10—O3	104.5 (2)
F2—C5—C3	110.7 (2)	O4—C10—C12	110.3 (3)
F1—C5—C3	109.6 (3)	O3—C10—C12	108.7 (3)
F2—C5—C6	107.8 (2)	O4—C10—C11	110.7 (3)
F1—C5—C6	111.1 (2)	O3—C10—C11	109.9 (3)
C3—C5—C6	112.0 (3)	C12—C10—C11	112.3 (3)
O2—C6—C7	107.65 (18)	C10—C11—H11A	109.5
O2—C6—C5	108.6 (2)	C10—C11—H11B	109.5
C7—C6—C5	114.4 (2)	H11A—C11—H11B	109.5
O2—C6—H6	106.4 (16)	C10—C11—H11C	109.5
C7—C6—H6	112.1 (17)	H11A—C11—H11C	109.5
C5—C6—H6	107.4 (16)	H11B—C11—H11C	109.5
C6—C7—C8	112.3 (2)	C10—C12—H12A	109.5
C6—C7—H7A	109.1	C10—C12—H12B	109.5
C8—C7—H7A	109.1	H12A—C12—H12B	109.5
C6—C7—H7B	109.1	C10—C12—H12C	109.5
C8—C7—H7B	109.1	H12A—C12—H12C	109.5
H7A—C7—H7B	107.9	H12B—C12—H12C	109.5
			
C6—O2—C1—O1	−168.5 (3)	O2—C6—C7—C8	−62.7 (3)
C6—O2—C1—C2	15.5 (4)	C5—C6—C7—C8	176.5 (2)
O1—C1—C2—C3	−163.5 (3)	C10—O3—C8—C7	−140.1 (2)
O2—C1—C2—C3	12.3 (5)	C10—O3—C8—C9	−17.9 (3)
C1—C2—C3—C5	−4.4 (5)	C6—C7—C8—O3	−59.2 (3)
C2—C3—C5—F2	−148.5 (3)	C6—C7—C8—C9	−174.8 (2)
C2—C3—C5—F1	95.6 (3)	C10—O4—C9—C8	21.3 (3)
C2—C3—C5—C6	−28.1 (4)	O3—C8—C9—O4	−2.0 (3)
C1—O2—C6—C7	−170.7 (2)	C7—C8—C9—O4	116.0 (3)
C1—O2—C6—C5	−46.3 (3)	C9—O4—C10—O3	−32.7 (3)
F2—C5—C6—O2	173.1 (2)	C9—O4—C10—C12	−149.4 (3)
F1—C5—C6—O2	−71.9 (3)	C9—O4—C10—C11	85.6 (3)
C3—C5—C6—O2	51.1 (3)	C8—O3—C10—O4	31.3 (3)
F2—C5—C6—C7	−66.6 (3)	C8—O3—C10—C12	149.1 (3)
F1—C5—C6—C7	48.3 (3)	C8—O3—C10—C11	−87.6 (3)
C3—C5—C6—C7	171.3 (2)		

### Hydrogen-bond geometry (Å, °)

**Table:**

*D*—H···*A*	*D*—H	H···*A*	*D*···*A*	*D*—H···*A*
C8—H8···O1^i^	0.95 (3)	2.66 (3)	3.578 (4)	161 (2)
C6—H6···O1^ii^	0.96 (3)	2.44 (3)	3.318 (4)	151 (2)

Symmetry codes: (i) *x*−1/2, −*y*+1/2, −*z*+2; (ii) *x*+1/2, −*y*+1/2, −*z*+2.
